# N-terminal pro-brain natriuretic peptide and associated factors in the general working population: a baseline survey of the Uranosaki cohort study

**DOI:** 10.1038/s41598-017-06090-6

**Published:** 2017-07-19

**Authors:** Atsushi Tanaka, Hisako Yoshida, Atsushi Kawaguchi, Jun-ichi Oyama, Norihiko Kotooka, Shigeru Toyoda, Teruo Inoue, Masafumi Natsuaki, Koichi Node

**Affiliations:** 1 Department of Cardiovascular Medicine, Saga University, Saga, Japan; 2 Clinical Research Center, Saga University, Saga, Japan; 3 Department of Cardiovascular Medicine, Dokkyo Medical University, Mibu, Japan; 4 Department of Internal Medicine, Imari Matsuura Hospital, Imari, Japan

## Abstract

Few data on clinical characteristics associated with N-terminal pro-brain natriuretic peptide (NT-proBNP) or the clinical value of measuring NT-proBNP in the working population are available. The aim of the present study was to investigate the levels of NT-proBNP and their association with clinical variables in the Japanese general working population by using baseline data from the Uranosaki cohort study. In the study, the plasma concentration of NT-proBNP and some biomarkers were measured in addition to the standard health checkups at the workplace. Questionnaires regarding health-related quality of life (HR-QOL) were also completed. A total of 2140 participants were enrolled in the study. Plasma levels of NT-proBNP were positively associated with age, female sex, systolic blood pressure, pulse pressure, prevalent hypertension, smoking habit, high-density lipoprotein cholesterol (HDL-C), and prevalent proteinuria, and negatively associated with body mass index, lipid profiles except HDL-C, uric acid, renal function, and hemoglobin. Both the plasma concentration of high-molecular weight adiponectin and that of high-sensitivity troponin T were positively and independently associated with NT-proBNP. In addition, the HR-QOL score regarding sleep disorder was independently associated with NT-proBNP. Thus, we have obtained evidence that the plasma NT-proBNP is affected by several clinical variables in the general working population.

## Introduction

Brain natriuretic peptide (BNP) and N-terminal pro-brain natriuretic peptide (NT-proBNP) are cardiac hormones secreted by ventricular myocardium in response to increased ventricular wall pressure and volume overload^1, 2^. Plasma concentrations of these hormones are powerful tools for the diagnosis and management of patients with acute or chronic heart failure (CHF) in the routine clinical settings. Several studies found that elevated levels of these hormones were strongly associated with poor outcomes in patients with heart failure and/or ischemic heart disease (IHD)^3–5^. Moreover, it is reported that BNP/NT-proBNP-guided screening and therapy is useful for preventing development of cardiac events including heart failure^6, 7^.

Previous community-based prospective studies also indicated that elevated levels of these hormones were significant prognostic risk factors for cardiovascular disease (CVD) and mortality beyond established risk factors^8–11^. In the Hisayama study in Japan^11^, the risk of CVD in individuals with relatively low levels of NT-proBNP (55–124 pg/mL) was 1.9-fold higher than in those with the lowest levels (<55 pg/mL) independent of conventional risk factors. Recent evidence suggested that even lower levels of NT-proBNP may contribute to future CVD and mortality^11–13^. Meanwhile, a previous report described inverse relationships between NT-pro BNP level and metabolic CV risk factors and the metabolic syndrome (MetS)^14^.

What connects NT-proBNP to the future development of CVD and worsened mortality? To prevent adverse clinical events, it is pertinent to determine its relation to other risk factors and identify individuals in the general population who are at preclinical or high CV risk with elevated levels of NT-proBNP. However, few data from large-scale and long-term observations in the Japanese general population are available, and the clinical significance of measuring NT-proBNP is unclear. In addition, it is known that worsened health-related quality of life (HR-QOL) state is bi-directionally associated with CVD, including chronic heart failure^15–17^. However, little is known about such association in the general population. If such association exists at preclinical level, early detection and medical intervention would help to prevent future development of CVD.

In the present study, it was therefore our aim to investigate the level of NT-proBNP and its associations with clinical variables in the general working population. In addition, we sought to address the relation between NT-proBNP and some HR-QOL scores. Although the ultimate goal is to assess the longer-term prognostic value of NT-proBNP, the present study is a baseline survey of the project.

## Methods

### Overall study design and population

The present study was a baseline survey of the Uranosaki cohort study, which was designed as a prospective study to evaluate the prognostic value of NT-proBNP concentration and its clinical associations with metabolic CV risk factors. The Uranosaki cohort study began in 2008 in Uranosaki Hospital (currently renamed as the Imari Matsuura Hospital), Imari, Saga, located in the north of Kyusyu Island in Japan. The study conformed to the policy of the Japanese government of that time, which implemented regular health checkups at the workplace to obtain information on the prevalence of MetS and prevent its development or progression for insured individuals in the working population^18^. In this health checkup system, each work office and company can outsource standard health checkups to the hospital. Then, in addition to the checkups in the workplace, some biomarkers including NT-proBNP were measured, and questionnaires regarding HR-QOL were completed. The protocol was approved by the Institutional Review Boards at both Saga University and Uranosaki Hospital. The study was conducted in full compliance with the Declaration of Helsinki and was carried out according to the Ethical Guidelines for Clinical Research established by the Ministry of Health, Labour, and Welfare in Japan. After acceptance of this study at each work office or company, informed consent was obtained from each participant prior to enrollment. The eligible participants were health checkup examinees at their workplaces who agreed to the study and had given written consent. Individuals belonging to a work office or company where the study was not permitted and individuals who did not give informed consent were excluded.

### Clinical evaluation and laboratory measurements

At the examination, height, weight, and waist circumference were measured. Body mass index (BMI) was calculated, and obesity was defined as a BMI level ≥25 kg/m^2^. Blood pressure (BP) was obtained twice using an automated sphygmomanometer in the sitting position after a few minutes’ resting. The average value from two measurements was used, and hypertension was defined as systolic BP ≥ 140 mm Hg, diastolic BP ≥ 90 mm Hg, and/or receiving treatment with anti-hypertensive agents. Blood samples were obtained for routine clinical chemistry, such as liver enzymes, lipid profile, glucose level, and kidney function. MetS was defined as visceral obesity and metabolic disorders based on the diagnostic criteria for Japanese subjects described elsewhere^19^. Estimated glomerular filtration rate (eGFR) was calculated using modified equations for Japanese^20^. Residual serum was immediately frozen and stored at −80 °C for measurement of biomarkers including NT-proBNP, high-molecular weight (HMW)-adiponectin, and high-sensitivity troponin T (hsTnT). These biomarkers were measured in single batches at central laboratories: Roche Diagnostics Ltd, Fukuoka for NT-proBNP and hsTnT, and FUJIREBIO Inc., Tokyo for HMW-adiponectin. The levels of NT-proBNP and hsTnT were measured by an electrochemiluminescence immunoassay (Roche, Basel, Switzerland), as described elsewhere^21^. The level of HMW-adiponectin was measured specifically using a sandwich ELISA kit based on a monoclonal antibody to human HMW-adiponectin, IH7^22, 23^. Proteinuria was diagnosed by the test paper method using spot urine.

Each participant completed a self-administered questionnaire regarding health-related QOL, such as EuroQoL 5 dimensions (EQ-5D), Epworth Sleepiness Scale (ESS), and Pittsburgh Sleep Quality Index (PSQI). Decreased HR-QOL was defined as an EQ-5D level <1. Abnormal sleepiness was defined as an ESS level ≥11. Sleep disorder was defined as a PSQI level ≥5.5.

### Analysis protocol

The present study includes a 2-step flow of analysis protocol (Supplementary Fig. 1). In the early part of the study, participants with any missing data were excluded from the analyses (1st step). In the latter part, exploring the relationship between NT-proBNP and HR-QOL among the 2140 participants, we analyzed each HR-QOL score individually using subgroups provided by the participants who were without missing individual HR-QOL data (2nd step).

### Statistical analysis

Continuous variables were summarized as the mean ± standard deviation for a normal distribution, or median [IQR] for a skewed distribution. To compare baseline characteristics between male and female, independent two-tailed t-test, Mann-Whitney U test, and chi-square test or Fisher’s exact test were used for the analysis of continuous and categorical variables, respectively, as appropriate. We used analysis of variance (ANOVA) to compare the means of each variable between the groups stratified by NT-proBNP levels. Variables with a skewed distribution, such as HMV-adiponectin, hsTnT, and triglyceride, were used after transformed to logarithms for ANOVA. We performed multivariable regression analyses to confirm the effect of other variables on logarithmic NT-proBNP. In the second step analysis, we compared NT-proBNP levels by each of the HR-QOL index levels. In addition, heterogeneity of the relationship with sex was evaluated by adding an interaction term. All statistical analyses were performed with JMP version 11.0 software (SAS Institute, Inc., Cary, NC, USA). A *P* value < 0.05 was considered statistically significant.

### Data Availability

The datasets generated during and/or analyzed during the current study are available from the corresponding author on reasonable request.

## Results

### Participant characteristics

**Figure 1:**
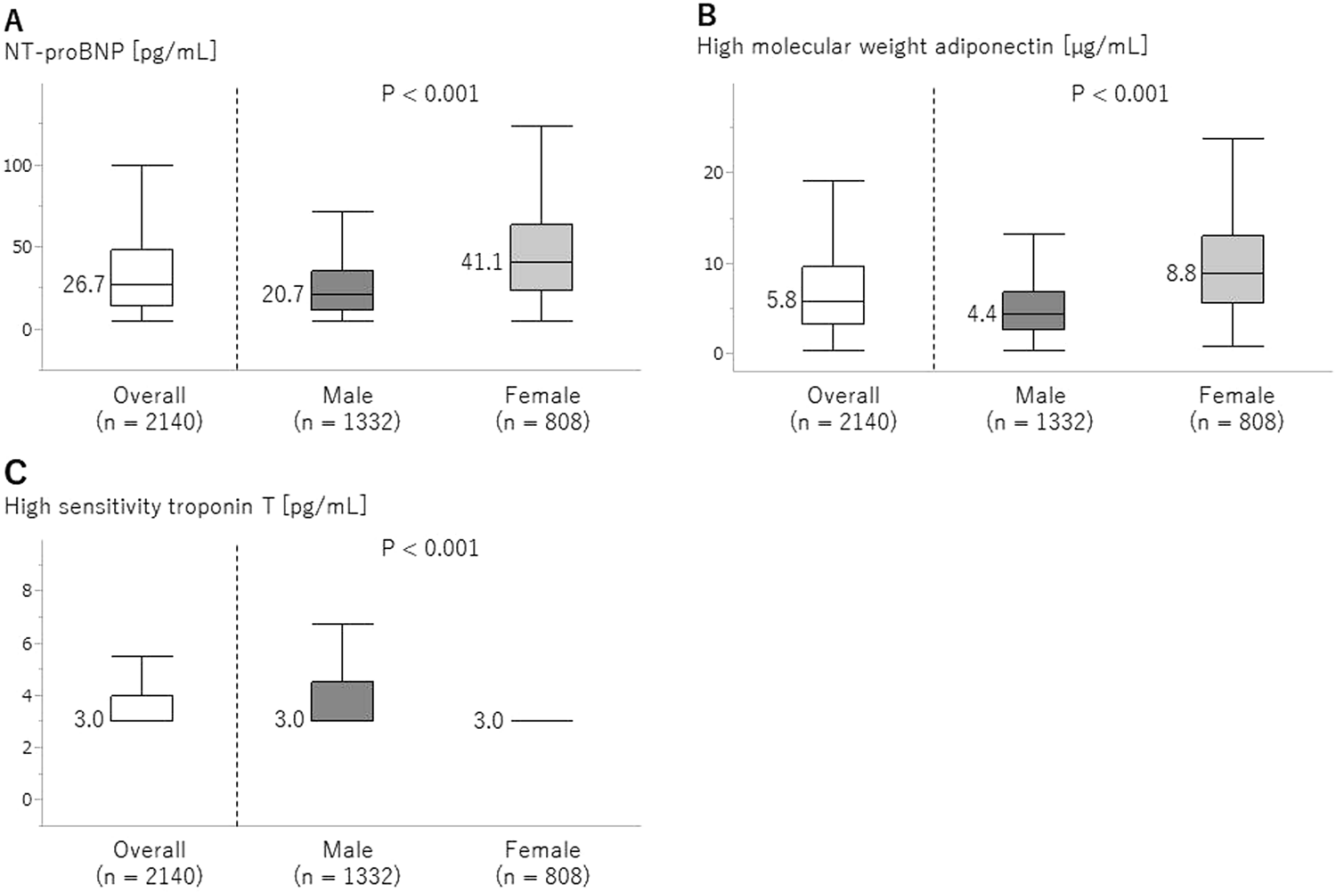
Plasma concentrations of NT-proBNP (**A**), HMW-adiponectin (**B**), and hsTnT (**C**) in the total cohort and by sex. Comparisons between male and female were performed by Mann-Whitney U test.

Of the 4996 subjects enrolled in the study, 2856 subjects with missing data were excluded from the analyses (Supplementary Fig. 1). The characteristics of the 2140 participants are shown in Table 1. Ages were 49.9 ± 8.2 years (mean ± SD), and 808 subjects (37.8%) were females. Females had less obesity, hypertension, MetS, current smoking habit, and proteinuria. In females, the levels of total cholesterol (TC) and high-density lipoprotein cholesterol (HDL-C) were higher, and those of triglyceride, uric acid, fasting blood sugar (FBS), and hemoglobin were lower than in male participants. The serum levels of NT-proBNP and HMW-adiponectin were significantly higher in females than in males (Fig. 1). By contrast, the serum levels of hsTnT in females were lower than in males.

**Table 1:** Baseline characteristics.

	Total (n = 2140)	Male (n = 1332)	Female (n = 808)	P-value (Male vs. Female)
Age; yrs	49.9 ± 8.2	49.6 ± 8.3	50.6 ± 7.9	0.005
Distribution; n (%)				0.006
<40	298 (13.9)	207 (15.5)	91 (11.3)	
40 to 49	666 (31.1)	421 (31.6)	245 (30.3)	
50 to 59	957 (44.7)	564 (42.3)	393 (48.6)	
60 to 69	205 (9.6)	134 (10.1)	71 (8.8)	
≥70	14 (0.7)	6 (0.5)	8 (1.0)	
BMI; kg/m^2^	22.9 ± 3.2	23.2 ± 3.1	22.3 ± 3.3	<0.001
Waist circumference; cm	81.2 ± 8.9	82.7 ± 8.6	78.7 ± 8.7	<0.001
Obesity; n (%)	501 (23.4)	351 (26.4)	150 (18.6)	<0.001
Systolic blood pressure; mm Hg	125.9 ± 15.7	127.1 ± 15.1	123.9 ± 16.5	<0.001
Diastolic blood pressure; mm Hg	79.0 ± 10.5	80.8 ± 10.2	76.1 ± 10.4	<0.001
Pulse pressure; mm Hg	46.9 ± 10.5	46.3 ± 10.0	47.8 ± 11.3	0.002
Hypertension; n (%)	634 (29.6)	427 (32.1)	207 (25.6)	0.002
Metabolic syndrome; n (%)	278 (13.0)	244 (18.3)	34 (4.2)	<0.001
Current smoking; n (%)	761 (35.6)	657 (49.3)	104 (12.9)	<0.001
Total cholesterol; mg/dL	205.2 ± 34.1	202.8 ± 33.8	209.2 ± 34.3	<0.001
Triglyceride; mg/dL	83 [60, 123]	95 [68, 143]	69 [52, 94]	<0.001
HDL-cholesterol; mg/dL	65.4 ± 17.0	61.5 ± 16.5	72.0 ± 15.7	<0.001
LDL-cholesterol; mg/dL	125.7 ± 32.4	125.5 ± 32.6	126.1 ± 32.1	0.670
Uric acid, mg/dL	5.4 ± 1.5	6.0 ± 1.4	4.4 ± 1.1	<0.001
Fasting blood sugar, mg/dL	100.3 ± 17.5	102.7 ± 17.9	96.3 ± 16.1	<0.001
eGFR; mL/min/1.73 m^2^	78.6 ± 14.2	77.8 ± 14.2	79.9 ± 14.2	0.001
Proteinuria; n (%)	73 (3.4)	59 (4.4)	14 (1.7)	<0.001
Hemoglobin; g/dL	14.3 ± 1.6	15.1 ± 1.1	12.9 ± 1.3	<0.001
NT-proBNP; pg/mL	26.7 [14.6, 48.6]	20.7 [11.9, 35.7]	41.1 [23.6, 63.6]	<0.001
High molecular weight adiponectin; µg/mL	5.8 [3.2, 9.6]	4.4 [2.6, 6.9]	8.8 [5.6, 13.0]	<0.001
High sensitivity troponin T; pg/mL	3.0 [3.0, 4.0]	3.0 [3.0, 4.5]	3.0 [3.0, 3.0]	<0.001

Data are shown as Mean ± SD, Median [IQR], or n (%).

Each *P* value was calculated t-test for variables with normal distribution and Mann-Whitney U test for skewed distribution. BMI = body mass index, eGFR = estimated glomerular filtration ratio, HDL = high-density lipoprotein,

hsTnT = high-sensitivity troponin T, LDL = low-density lipoprotein, NT-proBNP = N-terminal pro-brain natriuretic peptide.

## Distribution of NT-proBNP levels

**Figure 2:**
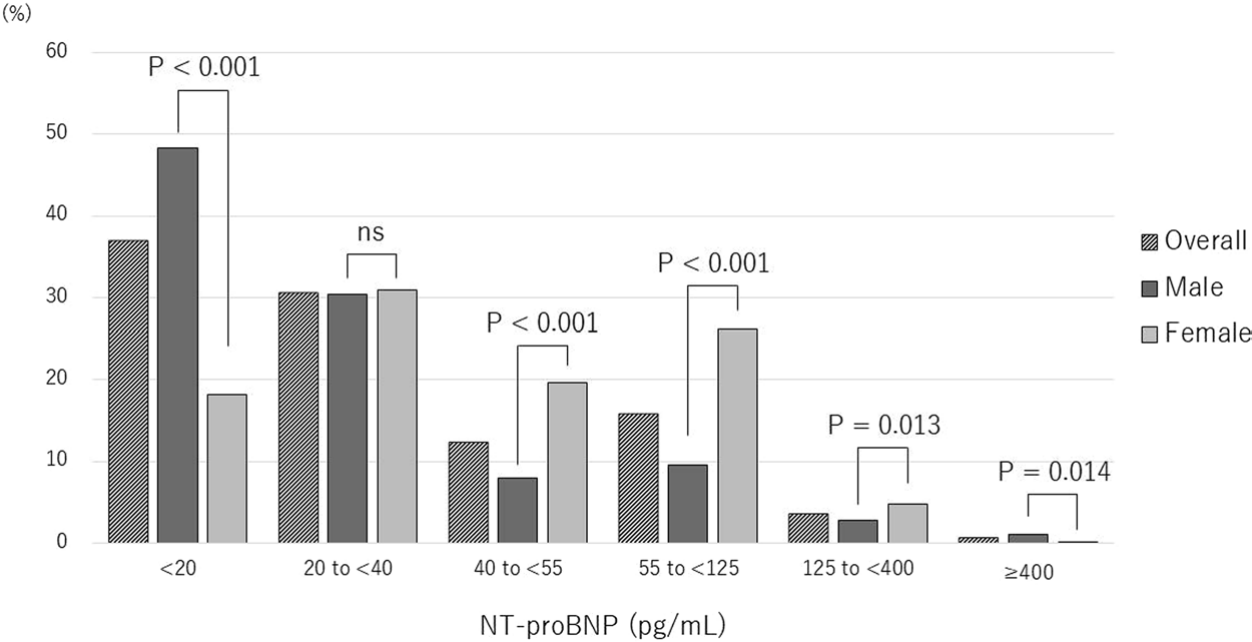
Distribution of NT-proBNP concentration. A sex difference was evident in each category except 20 to <40 pg/mL.

About 20% of the participants had NT-proBNP levels ≥55 pg/mL. Although there was no significant sex difference in the prevalence of participants with NT-proBNP 20 to <40 pg/mL, significant sex differences were observed in the other categories (Fig. 2). The percentage of subjects with NT-proBNP over 55 pg/mL was significantly lower in males than in females (15.3% vs. 45.3%, P < 0.001).

### Risk factors according to NT-proBNP level

Table 2 (all subjects) and Supplementary Table 1 (by sex) show mean values or frequencies of possible risk factors according to the stratified NT-proBNP levels (<40, 40 to <55, 55 to <125, or ≥125 pg/mL). The mean values of age, systolic BP, pulse pressure, and hsTnT, and the frequencies of hypertension and proteinuria increased with increasing NT-proBNP level, while the mean values of TC, low-density lipoprotein-cholesterol (LDL-C), and hemoglobin decreased with increasing NT-proBNP level. When divided by sex, the mean values of BMI and the frequency of obesity were lower in the second (40 to <55 pg/mL) and third (55 to <125 pg/mL) subgroups in the female subgroup, but such factors contributed less in males. The mean values of systolic and diastolic BP, HDL-C, FBS, and HMW-adiponectin and the frequency of proteinuria rose with increasing NT-proBNP level, while the mean value of eGFR declined, in males but not in females (Supplementary Table 1).

**Table 2:** Factors correlated with NT-proBNP levels by ANOVA.

	NT-proBNP				P-value
	<40 pg/mL (n = 1445)	40 to<55 pg/mL (n = 266)	55 to<125 pg/mL (n = 338)	≥ 125 pg/mL (n = 91)	
NT-proBNP; pg/mL	18.5 [11.3, 27.1]	46.9 [43.7, 50.7]	74.9 [63.8, 88.6]	181.4 [141.7, 298.6]	<0.001
Male sex; n(%)	1048 (72.5)	107 (40.2)	126 (37.3)	51 (56.0)	<0.001
Age; yrs	48.7 ± 8	51.7 ± 7.7	52.3 ± 7.9	55.0 ± 8.6	<0.001
Distribution; n (%)					
<40	249 (17.2)	21 (7.9)	23 (6.8)	5 (5.5)	<0.001
40 to 49	476 (32.9)	77 (29.0)	95 (28.1)	18 (19,8)	
50 to 59	605 (41.9)	136 (51.1)	168 (49.7)	48 (52.8)	
60 to 69	113 (7.8)	28 (10.5)	49 (14.5)	15 (16.5)	
≥70	2 (0.1)	4 (1.5)	3 (0.9)	5 (5.5)	
BMI; kg/m^2^	23.1 ± 3.1	22.4 ± 3.4	22.4 ± 3.3	22.4 ± 3.7	<0.001
Waist circumference; cm	81.8 ± 8.6	80.2 ± 9.1	79.8 ± 9.6	80.1 ± 9.0	<0.001
Obesity; n (%)	365 (25.3)	49 (18.4)	67 (19.8)	20 (22.0)	0.030
Systolic blood pressure; mm Hg	124.9 ± 14.1	125.3 ± 16.6	128.1 ± 18.2	135.8 ± 21.5	<0.001
Diastolic blood pressure; mm Hg	79.2 ± 9.8	77.8 ± 11.1	78.3 ± 12	82.6 ± 13.6	0.001
Pulse pressure; mm Hg	45.6 ± 9.6	47.6 ± 10.7	49.8 ± 11.8	53.2 ± 14.2	<0.001
Hypertension; n (%)	381 (26.4)	80 (30.1)	119 (35.2)	54 (59.3)	<0.001
Metabolic syndrome; n (%)	195 (13.5)	27 (10.2)	40 (11.8)	16 (17.6)	0.229
Current smoking; n (%)	553 (38.3)	81 (30.5)	99 (29.3)	28 (30.8)	0.002
Total cholesterol; mg/dL	206.2 ± 33.3	207.6 ± 34.8	202.3 ± 36.1	194.3 ± 34	0.003
Triglyceride; mg/dL	88 [62, 131]	77 [57, 106]	76 [54, 106]	82 [67, 115]	<0.001
HDL-cholesterol; mg/dL	63.8 ± 16.8	68.8 ± 17.6	69.5 ± 15.7	66.2 ± 19	<0.001
LDL-cholesterol; mg/dL	127.5 ± 32	126.2 ± 32.2	120.8 ± 34.1	114.0 ± 28.2	<0.001
Uric acid, mg/dL	5.6 ± 1.4	5.1 ± 1.5	5.0 ± 1.5	5.4 ± 1.6	<0.001
Fasting blood sugar, mg/dL	100.6 ± 16.4	98.7 ± 17.7	99.3 ± 19.5	104.3 ± 25.3	0.038
eGFR; mL/min/1.73 m^2^	79.2 ± 14	77.3 ± 14.3	77.8 ± 13.6	75.6 ± 18.1	0.018
Proteinuria; n (%)	44 (3.0)	6 (2.3)	13 (3.9)	10 (11.0)	<0.001
Hemoglobin; g/dL	14.6 ± 1.5	13.7 ± 1.5	13.5 ± 1.7	13.7 ± 1.8	<0.001
High molecular weight adiponectin; µg/mL	5.1 [2.9, 8.6]	7.3 [4.2, 11.1]	7.8 [4.6, 12.4]	6.9 [4.0, 12.2]	<0.001
High sensitivity troponin T; pg/mL	3 [3, 4]	3 [3, 4]	3 [3, 4]	5 [3, 12]	<0.001

Data are shown as Mean ± SD, Median [IQR], or n (%).

*P* values were calculated by ANOVA for variables with normal distribution and chi-square test for frequency of variable. In the analysis of skewed variables, *P* values were calculated after transfer to logarithms by ANOVA. Abbreviations, see Table 1.

### Factors correlated with NT-proBNP

**Figure 3:**
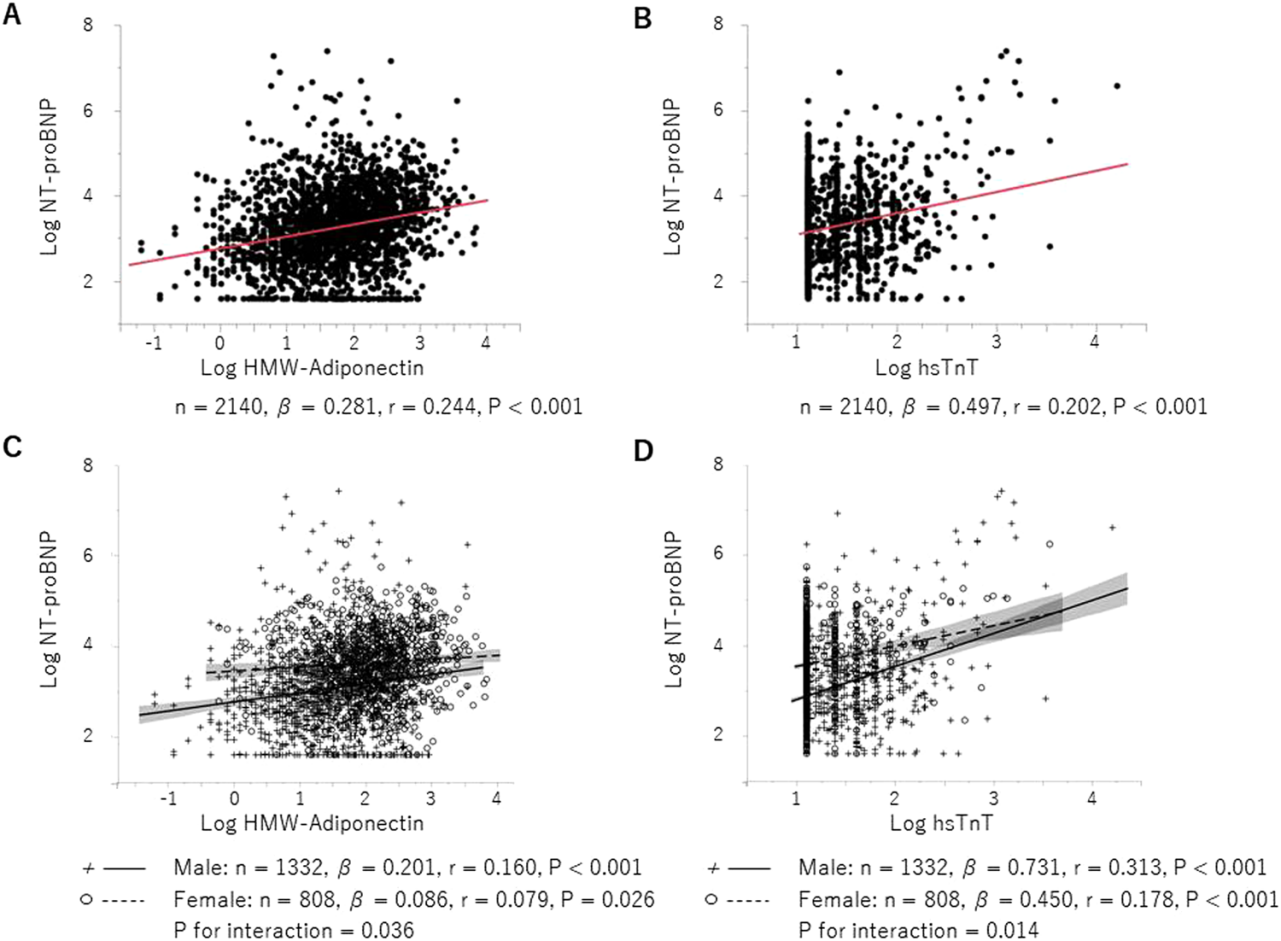
Correlations between NT-proBNP and HMW-adiponectin (**A** and **C**) or hsTnT (**B** and **D**). Significant sex differences were evident in the correlations (**C** and **D**).

Table 3 shows the factors correlated with log NT-proBNP. Univariate analysis demonstrated that age, systolic BP, pulse pressure, hypertension, smoking habit, HDL-C, and proteinuria were all positively correlated with log NT-proBNP, whereas male sex, BMI, waist circumference, obesity, lipid profiles except HDL-C, uric acid, eGFR, and hemoglobin were all negatively correlated with it. Among these factors, multivariate analysis revealed that age, systolic BP, smoking habit, and LDL-C were positively related to NT-proBNP, while male sex, BMI, TC, proteinuria, and hemoglobin were inversely related to it. Log HMW-adiponectin and log hsTnT were positively related to NT-proBNP in univariate and multivariate analyses, respectively (Table 3 and Fig. 3A,B). Furthermore, when stratified by sex, the degree of positive correlations in male was stronger than female, indicating sex differences in the associations of NT-proBNP with HMW-adiponectin and hsTnT (Fig. 3C,D and Supplementary Table 2). In multivariate analysis, age, smoking habit, and log hsTnT were positively associated with NT-proBNP, while TC and hemoglobin were negatively associated with NT-proBNP, irrespective of sex (Supplementary Table 2).

**Table 3:** Factors correlated with log NT-proBNP by adjusted regression analysis.

	Univariate		Multivariate	
	*β*	P-value	*β*	P-value
Age	0.031	<0.001	0.021	<0.001
Male sex	−0.572	<0.001	−0.193	<0.001
Body mass index	−0.037	<0.001	−0.041	0.002
Waist circumference	−0.013	<0.001	0.005	0.234
Obesity	−0.162	0.001	−0.037	0.224
Systolic blood pressure	0.008	<0.001	0.007	<0.001
Diastolic blood pressure	0.001	0.914		
Pulse pressure	0.018	<0.001	0.003	0.202
Hypertension	0.268	<0.001		
Metabolic syndrome	−0.002	0.937		
Smoking habit	0.169	<0.001	0.083	<0.001
Total cholesterol	−0.002	<0.001	−0.006	<0.001
Log Triglyceride	−0.26	<0.001		
HDL-cholesterol	0.008	<0.001		
LDL-cholesterol	−0.003	<0.001	0.004	0.040
Uric acid	−0.129	<0.001	0.003	0.071
Fasting blood sugar	−0.002	0.102		
eGFR	−0.004	0.005	−0.002	0.103
Proteinuria	0.255	0.018	−0.117	0.012
Hemoglobin	−0.189	<0.001	−0.116	<0.001
Log High molecular weight adiponectin	0.281	<0.001	0.075	0.003
Log High sensitivity troponin T	0.497	<0.001	0.468	<0.001

N = 2140. After the forward stepwise procedure for selection of variables, multivariable regression analysis was performed adjusted for age, sex, BMI, waist circumference, obesity, systolic blood pressure, pulse pressure, smoking habit, total cholesterol, LDL-cholesterol, uric acid, eGFR, proteinuria, hemoglobin, logarithmic HMW-adiponectin and logarithmic hsTnT. Abbreviations, see Table 1.

### Association between NT-proBNP and HR-QOL

**Figure 4:**
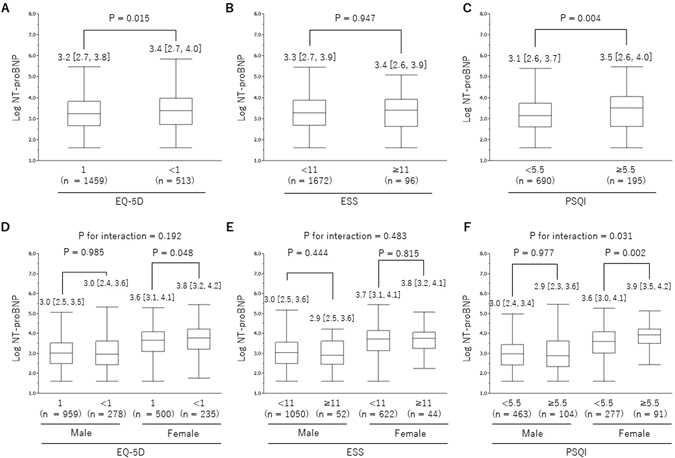
Influence of HR-QOL on NT-proBNP. EQ-5D in the total cohort (**A**) and by sex (**D**). ESS in the total cohort (**B**) and by sex (**E**). PSQI in the total cohort (**C**) and by sex (**F**). Each *P* value to compare between groups were calculated by Mann-Whitney U test.

Among the HR-QOL scores measured, plasma levels of NT-proBNP in participants with impaired HR-QOL status such as EQ-5D <1.0 or PSQI ≥5.5 were significantly higher than in those with normal scores, but this was not true of the ESS scores (Fig. 4A–C). Furthermore, these differences were more evident in females, especially in the PSQI (Fig. 4D–F). Stepwise multivariable regression analyses, which were performed to assess whether HR-QOL scores were associated with NT-proBNP, revealed that PSQI was significantly and positively associated with NT-proBNP after adjustment for age and sex (Table 4). The association between PSQI and NT-proBNP persisted after adjustment for other factors. When males and females were analyzed separately, a significant association after adjustment was observed only in the females. By contrast, no significant association between NT-proBNP and either EQ-5D or ESS was seen after adjustment (Supplementary Tables 3 and 4).

**Table 4:** Multivariate analysis of the association between NT-proBNP and PSQI.

	Overall (n = 885)						Male (n = 567)						Female (n = 318)					
	Model 1		Model 2		Model 3		Model 1		Model 2		Model 3		Model 1		Model 2		Model 3	
	*β*	P-value	*β*	P-value	*β*	P-value	*β*	P-value	*β*	P-value	*β*	P-value	*β*	P-value	*β*	P-value	*β*	P-value
PSQI	0.209	0.004	0.137	0.039	0.128	0.038	−0.003	0.977	0.015	0.867	0.024	0.775	0.294	0.002	0.296	0.002	0.269	0.002
Age			0.024	<0.001	0.022	<0.001			0.033	<0.001	0.027	<0.001			0.007	0.219	0.011	0.068
Male sex			−0.562	<0.001	−0.338	<0.001												
Body mass index					−0.028	0.003											−0.044	<0.001
Waist circumference																		
Obesity																		
Systolic blood pressure											0.008	0.020						
Diastolic blood pressure					0.004	0.164												
Pulse pressure					0.014	<0.001					0.012	0.019					0.006	0.188
Hypertension																	0.185	0.100
Metabolic syndrome					0.215	0.019												
Smoking habit					0.224	<0.001					0.174	0.009					0.292	0.011
Total cholesterol					−0.003	0.001					−0.003	0.003					−0.003	0.030
Log Triglyceride					−0.118	0.030											−0.187	0.054
HDL-cholesterol																		
LDL-cholesterol																		
Uric acid																		
Fasting blood sugar																		
eGFR																		
Proteinuria																	−0.522	0.197
Hemoglobin					−0.120	<0.001					−0.129	<0.001					−0.100	0.003
Log High molecular weight adiponectin					0.080	0.037					0.165	<0.001						
Log High sensitivity troponin T					0.208	0.007					0.144	0.124					0.306	0.023

In overall analyses, Model 1 was un-adjusted analyses, Model 2 was multivariable analyses which were adjusted for age and sex, and Model 3 was multivariable analysis which were adjusted for age, sex, BMI, diastolic blood pressure, pulse pressure, metabolic syndrome, smoking habit, total cholesterol, logarithmic triglyceride, hemoglobin, logarithmic high molecular weight adiponectin and logarithmic hsTnT. In subgroups of male and female subjects, Model 1 and 2 were adjusted for same factor with overall analysis. Model 3 in male group were systolic blood pressure, pulse pressure, smoking habit, total cholesterol, hemoglobin, logarithmic high molecular weight adiponectin and logarithmic hsTnT. In female group, Model 3 were age, BMI, pulse pressure, hypertension, smoking habit, total cholesterol, logarithmic triglyceride, proteinuria, hemoglobin and logarithmic hsTnT. The forward stepwise procedures were performed for variable selections. Abbreviations, see Table 1.

## Discussion

In this baseline survey, we investigated clinical associations between plasma levels of NT-proBNP and various factors, including metabolic/cardiac biomarkers, obtained from routine health checkups in the general working population. In addition, we performed exploratory analysis to assess the preclinical association between the levels of NT-proBNP and HR-QOL scores. To our knowledge this is the first report to comprehensively investigate the associations between NT-proBNP and various factors in a large cohort drawn from the Japanese working generation.

Recent reports showed that BNP/NT-proBNP-guided treatment can improve clinical outcomes and reduce the hospital stays of patients with CHF, leading to more cost-effective management of the condition^6, 7, 24–26^. In addition, it is well recognized that measurement of BNP/NT-proBNP concentration has greater accuracy for the prediction of CVD and mortality in patients with CHF and/or IHD^3, 27, 28^. In general population or community-based prospective studies, increased levels of BNP/NT-proBNP were associated with future adverse clinical outcomes and mortality beyond conventional risk factors^8, 9, 11, 29, 30^. These prospective studies showed that the risk of future development of CVD and mortality was increased in subjects even at the relatively low levels of BNP/NT-proBNP in the general population, indicating the clinical importance of early detection of subjects with subclinical conditions but at potential risk, and of risk stratification to improve future outcomes. Previous prospective studies often focused on subjects aged around 60 years at baseline^31^. Actually, clinical data on NT-proBNP in younger subjects were lacking, and factors correlated with NT-proBNP levels remained to be elucidated in this generation. Accordingly, we focused on the working generation and decided to evaluate NT-proBNP levels in addition to the usual health checkups in the workplace.

In line with previous reports^14, 32, 33^, NT-proBNP concentration was positively associated with age, female sex, and systolic blood pressure and negatively associated with BMI, MetS, TC, FBS, and hemoglobin on multivariate regression analysis. More recently, Hamada *et al*.^34^ also reported that age and serum hemoglobin concentration were strong determinants of plasma levels of atrial natriuretic peptides and BNP in Japanese younger healthy subjects aged 18–70 years (mean 34.1 years in males, and 38.3 years in females). The lower level of hemoglobin potentially affected cardiac function through a reduction of oxygen supply to the myocardium and impaired cardiac work efficiency, although no test of cardiac function using echocardiography was performed in the study. Thus, these results suggested that the determinants of the plasma NT-proBNP level were similar between Western and Japanese subjects across generation lines.

The inverse associations of plasma NT-proBNP with MetS and obesity might be attributable to the favorable actions of NT-proBNP on adiposity profile, such as lipolytic and fat mobilizing effects^35, 36^. This may in part account for the “obesity paradox” in which obese patients with CHF and/or IHD have more favorable clinical outcomes than lean patients^37–39^. Given the fact that decreased levels of NT-proBNP associate with increased body mass and accumulated visceral and liver fat, however, such protective actions of NT-proBNP on adipose tissue may be attenuated in subjects with obesity and MetS, possibly leading to systemic metabolic disturbance. Brutsaert *et al*.^40^ reported that higher NT-proBNP levels were associated with a decreased risk of developing diabetes, suggesting NT-proBNP-mediated favorable effects on certain metabolic pathways, including insulin resistance. However, once type 2 diabetes mellitus (T2DM) develops, an elevated level of NT-proBNP becomes a strong predictor of CVD and overall mortality in patients with T2DM^41^. Furthermore, Luchner *et al*.^42^ showed that prevalent diabetes at baseline, second only to aging, was strongly associated with elevation of BNP/NT-proBNP levels during 10 years of follow-up. Thus, it remains to be determined whether NT-proBNP directly contributes to the risk of CVD and mortality in subjects without apparent heart failure. Therefore, several contributing factors, such as MetS-relating parameters, underlying the NT-proBNP level should be taken into account when interpreting its value and defining appropriate cut-off values. Further long-term temporal observations of both NT-proBNP and associated factors are warranted.

Adiponectin is a major circulating adipocytokine secreted from adipose tissue and plays a role in regulating various metabolic pathways. A lower level of circulating adiponectin is associated with MetS, T2DM, and CVD^43–45^, suggesting CV protective actions of adiponectin. While, a higher level of adiponectin is associated with adverse clinical outcomes in patients with CHF and IHD, accompanied by elevated levels of NT-proBNP/BNP and decreased BMI^46, 47^. In fact, it is well accepted that NT-proBNP level is positively associated with circulating adiponectin level in both direct and indirect manners^48–50^. Circulating hsTnT is a biomarker derived from myocardium in response to myocardial injury. In the general population, the factors correlated with elevation of hsTnT are consistent with traditional CV risk factors^51, 52^. Recent studies have shown that the circulating hsTnT level could predict the future development of stroke, IHD, heart failure, and mortality in subjects without overt CVD^53–56^. Furthermore, simultaneous measurements of both NT-proBNP and hsTnT could improve prognostic predictive values in an additive manner^57–59^. Compared to NT-proBNP and adiponectin that have potentially protective actions on CV systems, hsTnT is a major biomarker of cardiac injury and presumably has no secondary action. Hitsumoto *et al*.^60^ recently reported that elevation of circulating hsTnT was associated with MetS-mediated dysfunction of the myocardial microcirculation and excess oxidative stress. In the present study, plasma concentrations of both HMW-adiponectin and hsTnT were independently related to NT-proBNP, despite the different biological nature of these biomarkers. Meanwhile, we found sex differences in the strengths of these associations, especially in the lower ranges of HMW-adiponectin and hsTnT. Although the explanations of these sex differences are unclear, they may arise in part from the actions of sex hormones or clinical baseline variables, such as metabolic parameters and hemoglobin level^61^.

Because HR-QOL is affected by various physical, mental, and social conditions, the precise mechanisms of the relation between HR-QOL and medical parameters are not easy to explain. However, to our knowledge, this is the first report to demonstrate associations between the plasma concentration of NT-proBNP and several HR-QOL scores, with clear sex differences in the associations. Interestingly, the level of PSQI was independently related to NT-proBNP. It is reported that overnight rostral fluid shift was associated with pathogenesis of sleep-disordered breathing, such as obstructive and central sleep apnea, in healthy men and men with heart failure^62, 63^. Patients with excess fluid volume or heart failure suffer from frequent sleep-disordered breathing^64^. Furthermore, sleep-disordered breathing itself causes heart failure via several pathophysiological mechanisms, such as hypoxemia, fluctuations in intrathoracic pressure, activation of sympathetic nervous system and inflammatory responses^65^. Thus, heart failure is associated closely and bi-directionally with sleep-disordered breathing. Therefore, current result of possible association between NT-proBNP levels and PSQI would be, in part, reasonable. However, several conflictions exist. First, in our study we could not assess the presence of heart failure and sleep-disordered breathing. Our result may reflect, in part, pathological association in preclinical levels of heart failure and sleep disorder. Second, to the best of our knowledge, there is no report investigating such association in women. In our study, there was association between NT-proBNP and PSQI in female, but not in male. In addition, PSQI is a comprehensive self-assessment of sleep quality, and is not necessarily suitable for evaluation of sleep-disordered breathing. Thus, the reasons for this sex difference observed was not determined in this cross-sectional study. Further study would be needed to assess the clinical significance of sex difference in the association of NT-proBNP and sleep quality in a general community population. It has been reported that the HR-QOL status of patients with CHF was impaired more than in some other chronic diseases^66^. Thus, measuring HR-QOL status may help us to assess physical and mental condition in individual patient with or suspected CVD. Moreover, the clinical importance of measuring patient-reported outcomes, including HR-QOL, in CV clinical trials has been recently discussed by international societies^67^. The clinical effectiveness of BNP/NT-proBNP-guided heart failure treatment on HR-QOL is currently controversial^68, 69^. Moreover, how HR-QOL influences NT-proBNP levels, or vice versa, and whether this relationship has prognostic value remain to be elucidated in the general population including the working generation. Therefore, a more detailed assessment of the clinical relationship between NT-proBNP levels and HR-QOL is warranted, as is also whether this relationship in people with and without overt CVD could impact on future clinical outcomes.

Because the present data are cross-sectional, they do not permit us to conclude there is a causal relationship between plasma NT-proBNP and the various risk factors. In addition, no clinical information regarding previous medical history and treatment status except hypertension were obtained in our study. Hence, we cannot exclude a possibility that the present cohort included subjects with diseases such as CHF and CVD, and accordingly our findings might partly reflect such conditions. Second, the present study had lots of excluded patients due to missing data. In the current health checkup at the workplace, it was difficult to avoid the missing data of examination and questionnaires. Third, the levels of hsTnT were much lower and under a measurement limit of hsTnT in the routine clinical settings. Hence, clinical significance of such level of hsTnT may be unclear. Further prospective observation would be needed to investigate whether such level of hsTnT in the working generation has impact on future cardiovascular outcomes. Last, because the data on HR-QOL were self-reported, they might be less objective and reproducible. It was for this reason that our exploratory analysis of the association between NT-proBNP and each HR-QOL was performed in the final part of the results, separated from the other analyses.

The present study shows that plasma NT-proBNP measured in the general working population is affected by multiple clinical variables, such as age, sex, blood pressure, metabolic CV parameters, and hemoglobin. Plasma NT-proBNP concentration was also associated with HMW-adiponectin, hsTnT, and some measures of HR-QOL status, with clear sex differences. Furthermore, self-reported sleep disorder as assessed by PSQI was independently associated with level of NT-proBNP in the general working population. These potential confounding factors should be taken into account when interpreting NT-proBNP values. At present, clinical evidence on whether and how NT-proBNP levels in the general working population should be managed is lacking, although previous studies demonstrated that a small elevation in the NT-proBNP level increased the risk of future CVD even in the general population^11, 31, 70^. Therefore, further clinical studies with long-term observations will be required to assess the clinical utility of measuring NT-proBNP in the general working population and the prognostic value of NT-proBNP for clinical outcomes.

## Electronic supplementary material

Supplementary Information
