# Supplementary material for: N-terminal pro-brain natriuretic peptide and associated factors in the general working population: a baseline survey of the Uranosaki cohort study
Source: Sci Rep. 2017 Jul 19;7:5810. doi: 10.1038/s41598-017-06090-6 (PMC5517578; doi:10.1038/s41598-017-06090-6)
Supplement: Supplementary file 1 — Supplementary Information [file 41598_2017_6090_MOESM1_ESM.pdf]

N-terminal pro-brain natriuretic peptide and associated factors in the general working population: a baseline survey of the Uranosaki cohort study

Atsushi Tanaka[1], Hisako Yoshida[2], Atsushi Kawaguchi[2], Jun-ichi Oyama[1], Norihiko Kotooka[1], Shigeru Toyoda[3], Teruo Inoue[3], Masafumi Natsuaki[4] & Koichi Node[1]

[1]Department of Cardiovascular Medicine, Saga University, Saga, Japan

[2]Clinical Research Center, Saga University, Saga, Japan

[3]Department of Cardiovascular Medicine, Dokkyo Medical University, Mibu, Japan

[4]Department of Internal Medicine, Imari Matsuura Hospital, Imari, Japan

Correspondence and requests for materials should be addressed to A.T. (email: tanakaa2@cc.saga-u.ac.jp) and K.N. (email: node@cc.saga-u.ac.jp)

Supplementary Figure 1. Participants' flowchart for analyses

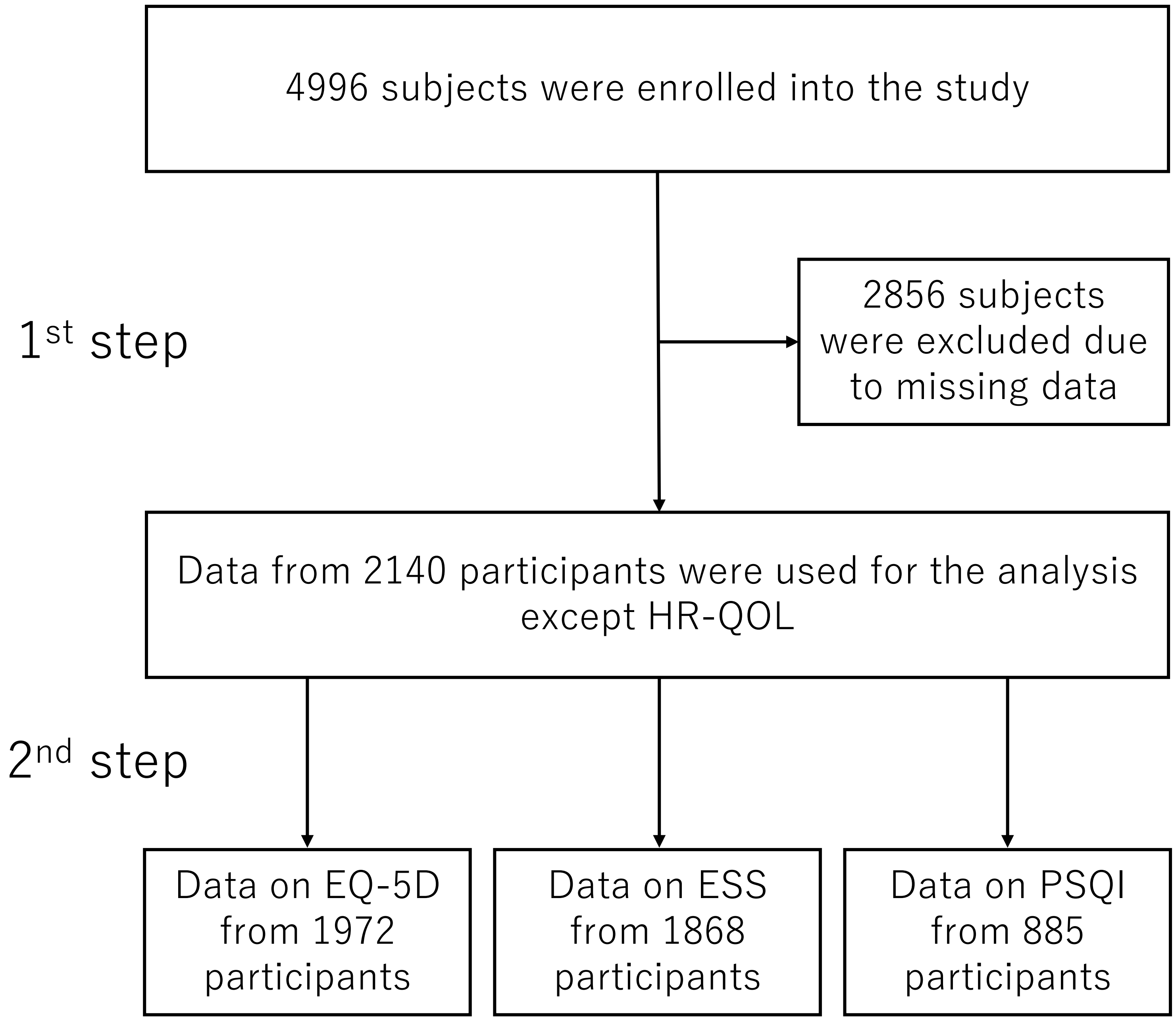

Supplementary Table 1

Factors correlated with NT-proBNP levels by sex

	Male					Female				
	NT-proBNP					NT-proBNP				
	<40 pg/mL (n = 1048)	40 to <55 pg/mL (n = 107)	55 to <125 pg/mL (n = 126)	≥125 pg/mL (n = 51)	P-value	<40 pg/mL (n = 397)	40 to <55 pg/mL (n = 159)	55 to <125 pg/mL (n = 212)	≥125 pg/mL (n = 40)	P-value
NT-proBNP; pg/mL	16.4 [10.0, 25.1]	46.1 [42.9, 50.5]	75.5 [63.2, 92.2]	213.8 [156.0, 516.3]	<0.001	23.5 [16.0, 31.4]	47.7 [43.9, 50.8]	74.7 [64.0, 87.8]	156.3 [135.6, 183.0]	<0.001
Age; yrs	48.4 ± 8.1	53.2 ± 8.2	53.7 ± 7.3	55.2 ± 7.7	<0.001	49.6 ± 7.7	50.7 ± 7.3	51.4 ± 8.1	54.8 ± 9.8	<0.001
Distribution; n (%)										
<40	196 (18.7)	7 (6.5)	1 (0.8)	3 (5.9)	<0.001	53 (13.4)	14 (8.8)	22 (10.4)	2 (5.0)	<0.001
40 to 49	349 (33.3)	25 (23.4)	30 (31.8)	8 (13.7)		127 (32.0)	52 (32.7)	55 (25.9)	11 (27.5)	
50 to 59	422 (40.3)	55 (51.4)	56 (44.4)	31 (60.8)		183 (46.1)	81 (50.9)	112 (52.8)	17 (42.5)	
60 to 69	80 (7.6)	17 (15.9)	28 (22.2)	9 (17.7)		33 (8.3)	11 (6.9)	21 (9.9)	6 (15.0)	
≥70	1 (0.1)	3 (2.8)	1 (0.8)	1 (2.0)		1 (0.3)	1 (0.6)	2 (0.9)	4 (10.0)	
BMI; kg/m^2	23.3 ± 3.0	23.1 ± 3.7	23.2 ± 3.6	22.6 ± 3.6	0.475	22.7 ± 3.3	21.9 ± 3.0	21.9 ± 3.1	22.1 ± 3.8	0.013
Waist circumference; cm	82.7 ± 8.3	83.1 ± 9.7	82.9 ± 9.9	81.6 ± 9.3	0.801	79.3 ± 8.9	78.3 ± 8.1	77.9 ± 9.0	78 ± 8.2	0.258
Obesity; n (%)	275 (26.2)	26 (24.3)	38 (30.2)	12 (23.5)	0.708	90 (22.7)	23 (14.5)	29 (13.7)	8 (20.0)	0.022
Systolic blood pressure; mm Hg	125.5 ± 13.7	129.7 ± 16.1	132.8 ± 17.1	141.4 ± 21.6	<0.001	123.3 ± 15.1	122.4 ± 16.3	125.2 ± 18.3	128.6 ± 19.3	0.085
Diastolic blood pressure; mm Hg	80.3 ± 9.6	81.5 ± 11.3	82.7 ± 12	85.9 ± 13.3	<0.001	76.5 ± 9.7	75.2 ± 10.3	75.7 ± 11.2	78.3 ± 12.8	0.266
Pulse pressure; mm Hg	45.2 ± 9.3	48.2 ± 9.6	50.1 ± 10.8	55.5 ± 13.9	<0.001	46.8 ± 10.2	47.1 ± 11.4	49.6 ± 12.3	50.3 ± 14.2	0.011
Hypertension; n (%)	292 (27.9)	40 (37.4)	60 (47.6)	35 (38.6)	<0.001	89 (22.4)	40 (25.2)	59 (27.8)	19 (47.5)	0.005
Metabolic syndrome; n (%)	178 (17.0)	22 (20.6)	29 (23.0)	15 (29.4)	0.054	17 (4.3)	5 (3.1)	11 (5.2)	1 (2.5)	0.742
Current smoking; n (%)	514 (49.1)	50 (46.7)	67 (53.2)	26 (51.0)	0.771	39 (9.8)	31 (19.5)	32 (15.1)	2 (5.0)	0.005
Total cholesterol; mg/dL	204 ± 33.2	201.6 ± 35.9	198.3 ± 34.2	191.8 ± 36.5	0.027	211.8 ± 32.9	211.6 ± 33.6	204.7 ± 37.1	197.4 ± 30.7	0.009
Triglyceride; mg/dL	98 [68, 148]	93 [66, 147]	84 [66, 126]	94 [72, 137]	0.193	69 [52, 93]	68 [50, 96]	69 [50, 95]	74 [55, 102]	0.703
HDL-cholesterol; mg/dL	60.6 ± 16.1	62.8 ± 17.2	65.9 ± 16.5	65.4 ± 21.3	0.002	72.3 ± 15.6	72.9 ± 16.8	71.7 ± 14.9	67.1 ± 15.7	0.200
LDL-cholesterol; mg/dL	127.3 ± 32.2	122.9 ± 32.6	118.6 ± 34.3	110.8 ± 31.4	<0.001	128.1 ± 31.7	128.5 ± 31.9	122.2 ± 34	118 ± 23.5	0.042
Uric acid, mg/dL	6.0 ± 1.3	6.1 ± 1.5	6.0 ± 1.4	6.1 ± 1.5	0.992	4.5 ± 1.0	4.4 ± 0.9	4.4 ± 1.1	4.5 ± 1.3	0.727
Fasting blood sugar, mg/dL	102.2 ± 16.6	102.7 ± 19.8	104 ± 19.3	109.8 ± 30.7	0.024	96.2 ± 14.9	96 ± 15.7	96.5 ± 19.1	97.2 ± 13.1	0.974
eGFR; mL/min/1.73m^2	78.7 ± 14	76.3 ± 14.7	74.6 ± 12.8	71.8 ± 18.3	<0.001	80.6 ± 14.1	78 ± 14.1	79.7 ± 13.7	80.5 ± 16.8	0.271
Proteinuria; n (%)	37 (3.5)	2 (1.9)	10 (7.9)	10 (19.6)	<0.001	7 (1.8)	4 (2.5)	3 (1.4)	0 (0.0)	0.704
Hemoglobin; g/dL	15.2 ± 1	14.9 ± 1.1	14.8 ± 1.2	14.8 ± 1.4	<0.001	13.1 ± 1.3	12.9 ± 1.1	12.7 ± 1.5	12.4 ± 1.2	<0.001
High molecular weight adiponectin; μg/mL	4.2 [2.5, 6.7]	5 [3.0, 7.5]	5.3 [3.3, 9.4]	5.5 [3.1, 8.4]	<0.001	8.6 [5.4, 12.1]	9.2 [5.8, 13.1]	8.8 [5.7, 14.6]	9.4 [6.3, 16.1]	0.257
High sensitivity troponin T; pg/mL	3 [3, 4]	3 [3, 5]	4 [3, 5]	7 [3, 15]	<0.001	3 [3, 3]	3 [3, 3]	3 [3, 3]	3 [3, 7]	<0.001

Data are shown as Mean ± SD, Median [IQR], or n (%). Abbreviations, see Table 1.

Supplementary Table 2

Factors correlated with logarithmic NT-proBNP by adjusted regression analysis by sex

	Male (n = 1332)				Female (n = 808)			
	Univariate		Multivariate		Univariate		Multivariate	
	β	P-value	β	P-value	β	P-value	β	P-value
Age	0.038	<0.001	0.028	<0.001	0.013	<0.001	0.016	<0.001
Body mass index	-0.018	0.026			-0.038	<0.001	-0.039	<0.001
Waist circumference	-0.004	0.157			-0.010	0.001		
Obesity	-0.042	0.467			-0.236	<0.001		
Systolic blood pressure	0.015	<0.001			0.003	0.071		
Diastolic blood pressure	0.011	<0.001	0.010	<0.001	-0.001	0.699		
Pulse pressure	0.023	<0.001	0.015	<0.001	0.007	0.003		
Hypertension	0.400	<0.001			0.143	0.016	0.081	0.008
Metabolic syndrome	0.200	0.002			-0.013	0.921		
Smoking habit	0.029	0.564	0.073	0.001	0.135	0.083	0.108	0.004
Total cholesterol	-0.003	<0.001	-0.003	<0.001	-0.003	<0.001	-0.003	<0.001
Log Triglyceride	-0.129	0.004			-0.097	0.090		
HDL-cholesterol	0.006	<0.001			-0.002	0.332		
LDL-cholesterol	-0.004	<0.001			-0.002	0.004		
Uric acid	-0.028	0.139			-0.073	0.003		
Fasting blood sugar	0.003	0.048			-0.003	0.106		
eGFR	-0.008	<0.001			0.001	0.859		
Proteinuria	0.499	<0.001			-0.176	0.379		
Hemoglobin	-0.160	<0.001	-0.118	<0.001	-0.099	<0.001	-0.111	<0.001
Log High molecular weight adiponectin	0.201	<0.001	0.154	<0.001	0.086	0.026		
Log High sensitivity troponin T	0.731	<0.001	0.465	<0.001	0.450	<0.001	0.378	<0.001

A list of variables, see Table 3. Abbreviations, see Table 1.

Supplementary Table 3

Multivariate analysis of the association between NT-proBNP and EQ-5D

	Overall (n = 1972)						Male (n = 1237)						Female (n = 735)					
	Model 1		Model 2		Model 3		Model 1		Model 2		Model 3		Model 1		Model 2		Model 3	
	β	P-value	β	P-value	β	P-value	β	P-value	β	P-value	β	P-value	β	P-value	β	P-value	β	P-value
EQ-5D	0.114	0.015	0.022	0.613	0.012	0.765	-0.001	0.985	-0.014	0.811	-0.035	0.531	0.117	0.048	0.094	0.109	0.095	0.085
Age			0.029	<0.001	0.022	<0.001			0.037	<0.001	0.026	<0.001			0.013	<0.001	0.017	<0.001
Male sex			-0.533	<0.001	-0.362	<0.001												
Body mass index					-0.027	<0.001											-0.045	<0.001
Waist circumference																		
Obesity																		
Systolic blood pressure					0.010	<0.001											0.005	0.009
Diastolic blood pressure					-0.005	0.081					0.008	0.003						
Pulse pressure											0.015	<0.001						
Hypertension																		
Metabolic syndrome					0.219	0.001					0.099	0.160					0.244	0.104
Smoking habit					0.164	<0.001					0.170	<0.001					0.217	0.007
Total cholesterol					-0.007	<0.001					-0.004	<0.001					-0.011	0.009
Log Triglyceride																		
HDL-cholesterol					0.004	0.035					0.003	0.067					0.006	0.152
LDL-cholesterol					0.003	0.099											0.008	0.063
Uric acid																		
Fasting blood sugar					-0.003	0.019					-0.003	0.030					-0.003	0.102
eGFR					-0.002	0.160					-0.003	0.104						
Proteinuria					0.220	0.026					0.273	0.020						
Hemoglobin					-0.122	<0.001					-0.127	<0.001					-0.108	<0.001
Log High molecular weight adiponectin					0.063	0.016					0.129	<0.001						
Log High sensitivity troponin T					0.480	<0.001					0.468	<0.001					0.404	<0.001

A list of variables, see Table 4. Abbreviations, see Table 1.

Supplementary Table 4

Multivariate analysis of the association between NT-proBNP and ESS

	Overall (n = 1768)						Male (n = 1102)						Female (n = 666)					
	Model 1		Model 2		Model 3		Model 1		Model 2		Model 3		Model 1		Model 2		Model 3	
	β	P-value	β	P-value	β	P-value	β	P-value	β	P-value	β	P-value	β	P-value	β	P-value	β	P-value
ESS	0.006	0.947	-0.022	0.803	0.003	0.966	-0.099	0.444	-0.103	0.401	-0.060	0.598	0.027	0.815	0.050	0.671	0.027	0.805
Age			0.028	<0.001	0.023	<0.001			0.036	<0.001	0.026	<0.001			0.013	0.001	0.018	<0.001
Male sex			-0.534	<0.001	-0.351	<0.001												
Body mass index					-0.023	0.001											-0.041	<0.001
Waist circumference																		
Obesity																		
Systolic blood pressure					0.009	<0.001											0.005	0.009
Diastolic blood pressure					-0.003	0.223					0.008	0.005						
Pulse pressure											0.014	<0.001						
Hypertension																		
Metabolic syndrome					0.222	0.001					0.106	0.148					0.261	0.088
Smoking habit					0.158	<0.001					0.150	0.003					0.229	0.006
Total cholesterol					-0.003	<0.001					-0.003	<0.001					-0.003	0.003
Log Triglyceride					-0.077	0.054											-0.131	0.063
HDL-cholesterol											0.002	0.220						
LDL-cholesterol																		
Uric acid																		
Fasting blood sugar					-0.003	0.009					-0.003	0.034					-0.003	0.103
eGFR											-0.003	0.188						
Proteinuria					0.361	0.001					0.450	<0.001						
Hemoglobin					-0.114	<0.001					-0.122	<0.001					-0.101	<0.001
Log High molecular weight adiponectin					0.083	0.003					0.145	<0.001						
Log High sensitivity troponin T					0.440	<0.001					0.405	<0.001					0.432	<0.001

A list of variables, see Table 4. Abbreviations, see Table 1.
